# Extracellular Vesicles in Cardiovascular Diseases: Alternative Biomarker Sources, Therapeutic Agents, and Drug Delivery Carriers

**DOI:** 10.3390/ijms20133272

**Published:** 2019-07-03

**Authors:** Suet Yen Chong, Choon Keong Lee, Chenyuan Huang, Yi Hsuan Ou, Christopher J. Charles, Arthur Mark Richards, Yub Raj Neupane, Maria Vazquez Pavon, Olga Zharkova, Giorgia Pastorin, Jiong-Wei Wang

**Affiliations:** 1 Department of Surgery, Yong Loo Lin School of Medicine, National University of Singapore, Singapore 119228, Singapore; 2 Cardiovascular Research Institute (CVRI), National University Heart Centre Singapore (NUHCS), Singapore 117599, Singapore; 3 Department of Pharmacy, National University of Singapore, Singapore 117543, Singapore; 4 Department of Medicine, National University Hospital, Singapore 119228, Singapore; 5 Department of Physiology, Yong Loo Lin School of Medicine, National University of Singapore, Singapore 117593, Singapore

**Keywords:** extracellular vesicles, cardiovascular disease, biomarker, therapeutic, drug delivery, theranostics

## Abstract

Cardiovascular diseases (CVD) represent the leading cause of morbidity and mortality globally. The emerging role of extracellular vesicles (EVs) in intercellular communication has stimulated renewed interest in exploring the potential application of EVs as tools for diagnosis, prognosis, and therapy in CVD. The ubiquitous nature of EVs in biological fluids presents a technological advantage compared to current diagnostic tools by virtue of their notable stability. EV contents, such as proteins and microRNAs, represent specific signatures of cellular activation or injury. This feature positions EVs as an alternative source of biomarkers. Furthermore, their intrinsic activity and immunomodulatory properties offer EVs unique opportunities to act as therapeutic agents per se or to serve as drug delivery carriers by acting as miniaturized vehicles incorporating bioactive molecules. In this article, we aim to review the recent advances and applications of EV-based biomarkers and therapeutics. In addition, the potential of EVs as a drug delivery and theranostic platform for CVD will also be discussed.

## 1. Introduction

Cardiovascular disease (CVD) encompasses a wide spectrum of pathologies of the heart and blood vessels including peripheral vascular disease, coronary artery disease, cerebrovascular disease, ischemic heart disease, and heart failure (HF) [1]. CVD remains the highest cause of mortality globally, and it contributes to a grave socio-economic burden despite continued improvement in disease treatment and management [2]. Prognosis of CVD, especially myocardial infarction (MI), is poor with the majority of patients who survive their first MI having a high chance of recurrent MI or other complications, such as progressive HF, contributing to the high readmission rates and global disease burden [2].

Early detection of the onset of CVD can greatly improve the management and prognosis of the disease [3]. While the two widely clinically applied diagnostic markers, namely the cardiac troponins and cardiac natriuretic peptides, have revolutionized the diagnosis and management of CVD, especially for acute coronary syndromes (ACS) and HF, their limitations and flaws remain abundant and clinically significant. The high sensitivity of cardiac troponins I and T assays is offset by the poor diagnostic specificity for acute MI, and the plasma concentration of the B-type natriuretic peptides is often confounded by age and some non-CVD complications, such as renal dysfunction [4]. This imposes an urgent need for more refined diagnostic biomarkers, which can fill existing significant gaps, improve diagnostic certainty, and provide early risk stratification in CVD.

Extracellular vesicles (EVs) have emerged as an alternative source for biomarker discovery in various diseases, including CVD. Recent findings on their involvement in intercellular communication suggest that EVs may play pathophysiological roles in the progression of various CVD, including MI, ischemia-reperfusion (IR) injury and coronary artery disease [5,6,7]. It has been reported that EVs transfer molecules, such as inflammatory cytokines and microRNAs (miRNA), from injured cardiomyocytes to a wide spectrum of cell types, such as immune cells, fibroblasts, and endothelial cells, to regulate inflammation, angiogenesis, and resolution of the injured tissues [5,8]. Given their unique features (intrinsic bioactivities, immunomodulation, and sizes in the nanoscale), EVs have recently been proposed for therapeutics as well as drug delivery purposes.

## 2. Extracellular Vesicles: Origin, Types, and Characterizations

EVs are small anuclear vesicles with lipid bilayer membrane containing different species of lipids, metabolites, proteins as well as ribonucleic acids (RNA) and deoxyribonucleic acids (DNA). Most cell types in our body release EVs and almost all bodily fluids, such as blood, urine, seminal fluid, breast milk, and saliva, contain EVs [9,10,11,12,13]. The discovery of EVs can be traced back to as early as 1967 when Peter Wolf first described them as minute particulate material (platelet dust), which could be separated by ultracentrifugation [14]. Ironically, the first reported function of EVs was as a waste removal mechanism of the cell, and hence, EVs were considered to be the cell debris in the early 90s [15]. It took another decade to recognize EVs as bioactive mediators in cell-to-cell communication and their critical roles in both physiological and pathological conditions [16]. In fact, EVs can be taken up by various cells via endocytosis, membrane fusion, or phagocytosis, after which their contents can remain functional in the recipient cells [17]. In addition, the transmembrane proteins and receptors on the EV surface could also interact with the recipient cells through ligand–receptor interactions and result in biological effects beyond directing target of EVs to specific cell types [17].

The term EVs, as coined by the International Society of Extracellular Vesicles (ISEV), encompasses all extracellular membrane-enclosed vesicles [18,19,20]. The EV community has reached consensus in recent years that EVs could be classified into three main types based on their biogenesis [19,20]. Exosomes, the smallest EVs, have diameters ranging from 30 to 100 nm. They are generated intracellularly inside multivesicular bodies and released to the extracellular space by a mechanism known as endosomal sorting complex required for transport (ESCRT) [21,22,23]. Mid-sized EVs, which are formed through budding from the plasma membrane, with a diameter of 100 to 1000 nm, are known as microvesicles, microparticles or ectosomes. In this review, we will refer to this class of EVs as microvesicles. The largest EVs are known as apoptotic bodies, with a size range from 800 to 5000 nm, budding from the cells undergoing apoptosis [22]. To characterize EVs, numerous methods have been developed to isolate various types of EVs from a variety of sources. Several isolation methods, such as precipitation, differential ultracentrifugation, and separation based on a density gradient, are well established, showing the ability to achieve large scale isolation with a minimal degree of variability [24,25,26]. The merits and disadvantages of those methods have been well reviewed before [20,27].

**Figure 1:**
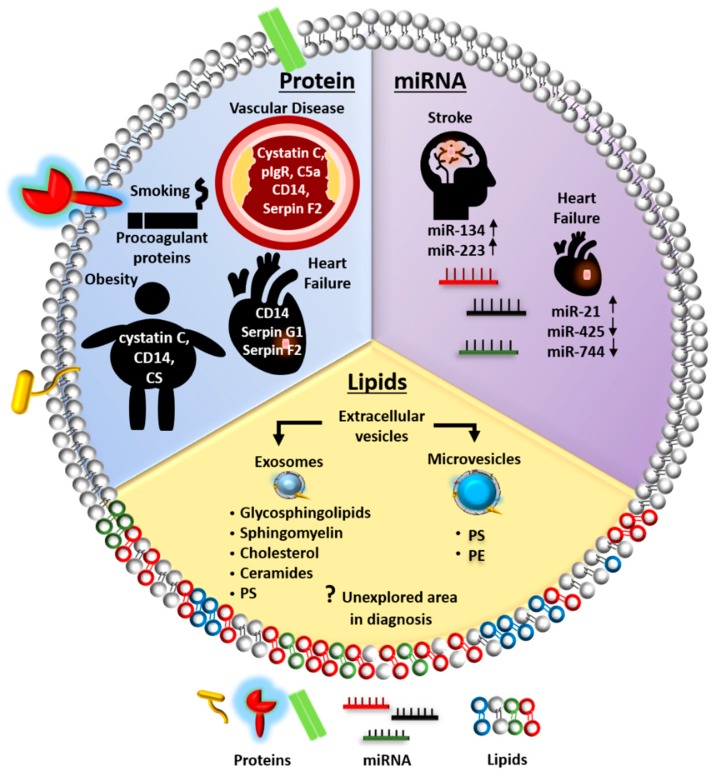
Schematic diagram illustrating EVs as an alternative source of biomarkers in cardiovascular disease (CVD). Extracellular vesicle (EV) proteins, such as CD14, cystatin C and coagulation-related proteins including Serpin G1 and Serpin F2, have been proposed as novel biomarkers for diagnosis and prognosis of heart failure, vascular disease, and stroke. Conventional CVD risk factors, such as obesity and smoking, have been associated with EV protein contents, including CD14, cystatin C, and procoagulant proteins. Certain EV miRNAs have also been identified as novel diagnostic and prognostic biomarkers for stroke and heart failure. Five EV miRNAs are listed as representatives. EV lipids, however, remain largely unexplored in the diagnosis or prognosis of CVD. CVD, cardiovascular diseases; CS, complement system; C5a, complement factor 5a; pIgR, polygenic immunoglobulin receptor; PS, phosphatidylserine; PE, phosphatidylethanolamine.

Apart from the size, EVs are often classified according to their surface proteins. These include mainly transmembrane or lipid-bound proteins: (1) Tetraspanins, such as CD63, CD9, and CD81; (2) Integrins and cell adhesion molecules; (3) Growth factor receptors; (4) Heterotrimeric G proteins; and (5) Phosphatidylserine binding proteins [28]. In particular, the transmembrane proteins have been widely used as a validation marker for EV identification since they are present in most EVs irrespective of their cellular origin [19,20]. Conversely, EV cargoes consist of a wide range of molecular compounds, such as nucleic acids, proteins, and lipids, which vary depending on the EV subtypes, cellular origin, and micro-environmental stimuli. For example, exosomes generally contain ESCRT proteins and accessory proteins, such as Alix and TSG101, which aid EV biogenesis, whereas microvesicles are abundant in proteins with posttranslational modifications, such as glycoproteins and phosphoproteins [29,30]. Furthermore, the content and amount of EVs released from cells can vary under different pathophysiological conditions [6]. Thus, EVs have been identified as a readily accessible source of biomarkers (Figure 1).

## 3. Extracellular Vesicle Contents in CVD

It is challenging to quantify and verify all the contents present in the EVs, as their contents are ever changing and always adapting to the local microenvironment. Different cell types also produce EVs with different contents due to distinctive cues from their microenvironment [31,32,33]. Nevertheless, the EV community is building up databases for EV contents, including protein, nucleic acid, and lipid entries, which are publicly accessible (http://www.exocarta.org/, http://student4.postech.ac.kr/evpedia2_xe/xe/, and http://microvesicles.org/). However, these database entries should be used with caution due to the diversity of the (patho-) physiological conditions in specific studies, EV nomenclature, and variations of EV isolation and characterization methods. The potential challenges and problem-solving recommendations have been thoroughly discussed in a European Society of Cardiology Working Group Position Paper [6].

In response to certain conditions, such as stress or injury, key components of EVs may be up- or down-regulated, presenting a unique signature that may serve as a potential diagnostic or prognostic biomarker for CVD (Figure 1 and Table 1) [34]. With recent advances in molecular technologies, several high throughput analytical approaches have been established to identify and quantify EV contents for diagnostic, prognostic, or therapeutic studies. Next generation deep sequencing and RT-qPCR are some of the techniques used to profile EV RNA or DNA [35]. Mass spectrometry (MS) based molecular characterization of EVs is utilized to identify multiple different species of lipids and proteins present in the EVs. Lipidomics and proteomics analyses are often paired with MS-based technologies, such as gas chromatography and liquid chromatography coupled to MS (GC-MS and LC-MS), to provide comprehensive and quantitative assessments of a large repertoire of lipid and protein species in EVs [36,37]. Once candidates are identified, multiplex assays on isolated EVs are commonly utilized to validate their biomarker value in clinical cohorts [38,39,40,41,42,43]. In this section, we have summarized recent clinical studies that have evaluated EV contents as potential diagnostic and prognostic biomarkers for various CVD (Table 1).

### 3.1. Extracellular Vesicle MicroRNA

Various species of RNA have been identified in EVs, including messenger RNA (mRNA), transfer RNA (tRNA), small interfering RNA (siRNA), long-non-coding RNA (lncRNA) and miRNA. Total RNA sequencing of EVs from human serum revealed that tRNA and miRNA made up approximately 15% of EV RNA [35]. Among the identified EV RNAs, miRNA has been closely associated with CVD [44,45,46].

miRNAs are single-stranded, small non-coding RNAs of 18 to 25 nucleotides in length, encoded by short inverted repeats within the genome [47]. miRNAs are secreted to the extracellular space through three main mechanisms: (1) direct excretion from the cell upon binding to RNA-binding proteins, (2) budding off the cells through microvesicles formation, or (3) packaged into multivesicular bodies and released from the cells as exosomes [48]. The main function of miRNA is to regulate gene expression via RNA-induced silencing complex (RISC) resulting in degradation or translational repression of targeted mRNA [49]. To date, more than 2000 miRNAs have been identified in the human genome, and most of the miRNAs in EVs are identified through small RNA deep sequencing or EV microarrays [50].

Many different EV miRNAs, such as miR-21, miR-126 and miR-146a, have been implicated in the pathogenesis of different stages of CVD, including atherosclerosis, cardiac hypertrophy, MI and HF (please see review [51]). A recent study proposes that EV miRNAs released following MI mediated the crosstalk between the injured heart and bone marrow, consequently inducing a systemic immune response to heart injury [52]. In line with this, EV miRNAs have been suggested to mediate ischemic preconditioning and postconditioning in cardiac injury [53,54,55]. Intriguingly, EV miRNAs are also involved in heart electrophysiology, mediating cardiac arrhythmia [55,56,57].

The potential application of EV miRNAs as biomarkers for CVD is less explored. Noticing EVs released to the circulation from the injured heart contained miRNAs, Deddens et al. have proposed EV miRNAs as early biomarkers for cardiac injury [58]. Given the protective nature of the lipid bilayer membrane, EVs could provide an enriched source of miRNAs compared to plasma or serum, enabling EV miRNAs to be detectable even with routine laboratory methods, such as qPCR [59,60,61]. In acute ischemic stroke patients, higher levels of miR-134 and miR-223 in circulating EVs correlated with the National Institutes of Health Stroke Scale (NIHSS) scores and brain infarct volume and consequently with a worse prognosis [59,60]. In HF, several EV miRNAs have been reported. In a small group of acute HF patients, higher levels of miR-21, and lower levels of miR-425 and miR-744 in EVs were reported [62]. A similar study identified EV miR-92b-5p as a potential diagnostic biomarker for acute HF [63]. In the Osaka Acute Coronary Insufficiency Study cohort, by extensive screening of miRNAs in serum EVs, Matsumoto et al. identified three p53-responsive miRNAs (miR-192, miR-194, and miR-34a) that could predict the development of HF a year after acute MI [64]. Apart from HF, several EV miRNAs were also reported in 40 patients with persistent atrial fibrillation, promising larger cohort studies on cardiac arrhythmia [57].

The utility of EV miRNAs as biomarkers in predicting the risk of developing CVD in individuals with high-risk factors, such as exposure to particulate matter, obesity, and diabetes, has also been explored. Exposure to particulate matter in air pollution has been shown to contribute to increased risk in developing CVD as well as cardiovascular morbidity and mortality, although the pathophysiological mechanisms remain elusive [65,66]. In humans, alteration of EV miRNA upon exposure to particulate matter correlates with increased blood pressure and coagulant state [67,68,69,70]. Obesity and related metabolic syndrome are important risk factors for CVD, while some EV miRNAs have been proposed as early biomarkers to predict CVD events in obese patients [71]. For ACS patients with severe artery blockage, coronary artery bypass graft (CABG) surgery may be recommended to improve the myocardial blood flow [72]. EVs are released to the circulation from the heart that undergoes CABG surgery [73]. Further studies suggest that some EV miRNAs may carry diagnostic information reflecting the extent of heart injury during CABG surgery or prognostic information predicting perioperative MI events following surgery [73,74,75]. Collectively, these promising findings need to be further validated in larger cohorts to assess the clinical application of EV miRNAs as novel biomarkers for CVD diagnosis and prognosis.

### 3.2. Extracellular Vesicle Proteins

Plasma protein levels of cardiac troponins and natriuretic peptides have long been established as diagnostic biomarkers in CVD, including ACS and HF [4,76]. However, those tests have their limitations under certain conditions. For instance, measuring cardiac troponins with high sensitivity troponin assays result in a loss of diagnostic specificity for acute MI, while measurement of natriuretic peptides in the accurate diagnosis of acute HF is often confounded by concurrent atrial fibrillation, renal dysfunction, and age. The clinically relevant shortcomings of those biomarkers have led to low reliability and accuracy for early detection and prognostic relevance in non-acute epidemiological risk stratification [4]. Most existing diagnostic protein biomarkers of CVD are reflections of the disease states and their concentrations peak for limited periods during or after disease onset. For instance, plasma troponin level is detectably elevated only for up to two weeks after MI [77]. In contrast to the time-sensitive and often labile nature of proteins in the circulation, proteins carried by EVs can be highly abundant and stable for extended periods [78]. The possibility to trace EVs to their cellular origin based on their distinctive surface markers allows them to be disease-specific, such as cardiomyocyte-derived EVs for CVD [7,79]. Proteomic profiling of EVs has identified many CVD-associated EV proteins in recent years (Table 1).

The first study associating EV protein levels with ACS was reported by de Hoog et al. (2013) [40]. Thirty-six proteins identified in serum EVs, using differential quantitative proteomics, were able to distinguish ACS patients from non-ACS subjects [40]. The concentrations of three selected EV proteins, namely polygenic immunoglobulin receptor (pIgR), cystatin C, and complement factor C5a, were significantly higher in the ACS-diagnosed patients compared with the non-ACS patients. In the setting of MI, Velez et al. identified an exclusive panel of EV proteins, which were differentially regulated in ST-elevation MI (STEMI) patients versus patients with stable coronary artery disease [80]. Among the 117 identified proteins, 102 proteins corresponded to 25 open-reading frames (ORF), and most of them are involved in the inflammatory pathway in response to cardiac injury. Notably, some identified proteins are also involved in the thrombogenesis pathway, indicating the involvement of EVs in the atherothrombotic events leading to MI [80].

The utility of EV proteins as prognostic biomarkers for CVD has also been investigated (Table 1). In a study of patients with a recent history of vascular disease or severe vascular risk factors, EV proteins were assessed for an association with secondary vascular events or mortality [41]. Differential proteomic profiling revealed 4 EV proteins (Cystatin C, Serpin G1, Serpin F2, and CD14) as potential prognostic markers, all of which are involved in vascular pathogenesis. After adjustment for conventional risk factors, EV protein levels of Cystatin C, Serpin F2, and CD14 were linearly related to increased risk of new vascular events, vascular mortality, and all-cause mortality [41]. In a dyspnoea patient cohort, EV proteins CD14, Serpin G1, and Serpin F2 correlated with the presence of HF [42]. Taken together, there is growing evidence that EV proteins may offer unique diagnostic and prognostic values for CVD.

EV proteins have also been associated with CVD risk factors, such as smoking and obesity. In an in vitro study with bronchial epithelium cells, EVs derived from epithelial cells that were exposed to cigarette smoke extract were shown to contain higher levels of pro-coagulant proteins, including tissue factor [83]. In fact, EVs present in human blood contain several coagulation-related proteins and therefore, may contribute to the increased risk of a thrombotic event resulting in MI or stroke [24,43,84]. Obesity has long been associated with an increased risk of CVD. However, no clinical biomarkers are available to delineate their association [85]. Interestingly, a study in patients with manifest arterial disease or CVD risk factors suggests that EV protein levels of cystatin C positively while CD14 negatively correlate with obesity and obesity-induced metabolic complications [81]. In line with this, 40 EV proteins, as demonstrated by 2D-DIGE and MS-based technologies, were altered in obese patients compared with age and gender-matched lean subjects [82]. The upregulation of the complement system (including complement C3, C4, and C9) and coagulation proteins (including fibrinogen, alpha-2-macroglobulin, kininogen-1, and prothrombin) in plasma EVs indicated a pro-inflammatory and pro-thrombotic state in obese patients. On the other hand, EV content of adiponectin, a protective protein hormone in obesity and CVD, was shown to be decreased in obese patients [82,86]. These studies demonstrate that EVs behave as “messengers”, shuffling between obesity and progression to CVD. However, it is not clear if the amount or composition of EV subtypes, or individual EV contents, is modified in the disease.

### 3.3. Extracellular Vesicle Lipids

All three classes of EVs comprise a lipid bilayer membrane with a similar composition to the plasma membrane, consisting mainly of phospholipids, glycolipids, and cholesterol [87]. Although they bear much resemblance, different EV subtypes have varying proportions or distribution of certain species of lipids [28,36]. Microvesicle membrane composition is highly similar to that of the plasma membrane due to its biogenesis, but the asymmetric distribution of phosphatidylserine (PS) and phosphatidylethanolamine (PE) is lost, resulting in an homogenous distribution of PS and PE across the bilayer membrane [88]. Exosome membrane is highly enriched in PS, glycosphingolipids, sphingomyelin, cholesterol, as well as ceramides [89]. The thickness of the lipid bilayer membrane of EVs is approximately 5 nm, contributing to two-thirds of the total mass of the smallest exosomes, which are as small as 30 nm in diameter [90]. The larger the size of EVs, the smaller the proportion of its total mass is contributed by its membrane lipids.

The abundance of lipids in EVs suggests that EV lipids may play a biological role beyond their structural function under physiological and certain pathophysiological conditions [91,92]. Ceramides, for instance, can be synthesized within the endosome and facilitate sorting and production of exosomes [89]. Ceramides are a class of bioactive lipids associated with apoptosis and inflammation. Under pathological conditions, EV ceramides may activate macrophages and promote their recruitment to the liver and therefore exacerbate hepatic inflammation [93]. In contrast, in the injured liver, EVs could transfer ceramide metabolic enzymes to produce sphingosine-1-phosphate (a metabolite of ceramides) in target hepatocytes and therefore promote liver repair and regeneration [94]. An interesting study by Khayrullin et al. indicated an association of EV ceramides with aging [95]. The authors isolated EVs from sera of healthy people and monkeys and found that EV level of a very long-chain C24:1 ceramide increases with age. Supplement of C24:1 ceramide into EVs from young people is able to induce cell senescence [95]. These findings suggest EV lipid composition can be modified by (patho-) physiological and environmental factors and meanwhile influence disease progression.

In the context of CVD, extensive studies have demonstrated the correlation of certain species of plasma lipids, in particular, ceramides, to the risk of developing CVD in human cohorts [96,97,98,99]. However, the role of EV lipids in CVD has not yet been reported, likely due to technical limitations. The technical advancement in EV isolation and the possible large-scale MS-based lipidomics profiling may shed light on the potential roles of EV lipids as diagnostic and prognostic biomarkers or therapeutic targets in CVD in the foreseeable future.

## 4. Intrinsic Activity of EVs: Potential Therapeutic Agents in CVD

The potential of EVs as a therapeutic agent arises from cell-based therapy preclinical studies and clinical trials. In these studies, stem cells or stem cell-derived cardiomyocytes were exogenously introduced into damaged heart tissue, however, only limited transplanted cells differentiated into new cardiomyocytes [100]. Given the poor engraftment and differentiation rates of the transplanted cells, the beneficial outcomes observed in some trials could not be explained by the newly generated cardiomyocytes. Instead, the transplanted cells may trigger endogenous repair mechanisms via paracrine factors, including EVs [6,101,102,103,104,105]. Apart from stem cell-derived EVs, the bioactivity of EVs of other cell origins will be discussed as well.

### 4.1. Extracellular Vesicles Derived from Stem Cells

Exosomes derived from mesenchymal stem cells (MSC) are the best characterized EVs, of which the intrinsic activity has been explored in the specific context of CVD. The earliest study of MSC-derived exosomes may be dated back to 2007 when Timmers et al. tried to identify the paracrine factors secreted in MSC conditioned medium, which protected the heart from IR injury in a pig model of MI [106]. Interestingly, the cardioprotective effects were attributed to large complexes >1000 kDa, with a size similar to viruses (100–220 nm). Later in 2010, the same group verified that those cardioprotective complexes were vesicles of 50 to 100 nm expressing exosome-surface markers (CD81, CD9, and Alix) [107,108]. Mechanistically, MSC-derived EVs protect the heart from tissue injury by decreasing oxidative stress, promoting cell viability, enhancing angiogenesis, or modulating immune response [103,109,110,111,112,113]. A very recent study showed that exosomes isolated from mouse bone marrow-derived MSC could change macrophage polarization, converting macrophages from pro-inflammatory to anti-inflammatory phenotype via EV miRNA-182 [114]. In a mouse model of IR injury, EV miRNA-182 inhibited the toll-like receptor 4 signaling pathway and consequently attenuated inflammatory response and alleviated heart injury [114].

Cardiosphere-derived cells, a type of stem cells residing within the heart, could also release EVs. Similar to MSC-derived EVs, EVs secreted by cardiosphere-derived cells exert powerful cardioprotective effects [115]. Induced pluripotent stem cells (iPS) have also been proposed for cell therapy promoting cardiac regeneration; however, the therapeutic application was impeded by their tumorigenicity [116,117,118]. A recent study explored the application of EVs derived from mouse cardiac fibroblast-generated iPS. In this study, EVs isolated from iPS were shown to prevent cell apoptosis in vitro and attenuated IR induced heart injury in vivo [119]. Another study suggested that EVs from iPS could transfer EV contents to recipient cardiac MSC, thus enhancing their proliferation and differentiation [120]. Despite limited data, these studies seem to provide a new direction for iPS-based therapy.

### 4.2. Extracellular Vesicles Derived from Cardiomyocytes and Cardiac Progenitor Cells

Cardiomyocytes also release EVs [52,121,122]. Instead of having intrinsic therapeutic effects, EVs released from cardiomyocytes under pathophysiological conditions may convey “danger signals” to other cells. In a hypoxia–normoxia cell injury model, cardiomyocytes released EVs containing heat shock protein 60 (HSP60), a ligand of toll-like receptor 4, which activates the innate immune response [121]. Similarly, cardiomyocytes released EVs carrying tumor necrosis factor-α (TNF-α) in response to hypoxia in vitro [123]. In a co-culture in vitro system, EVs released by diabetic cardiomyocytes could transfer miR-320 to their neighboring endothelial cells and inhibit the angiogenic activity of the recipient cells [124]. In vivo, EVs released from dying cardiomyocytes could convey the “danger signal” to remote bone marrow cells to attract them into the injured heart [52]. This is of particular interest since several toll-like receptors expressed in bone marrow-derived immune cells are recruited to the failing heart which contributed to exacerbated inflammation and poor resolution in MI or IR injury [125,126,127,128]. It is tempting to speculate that EVs from stressed cardiomyocytes mediate this process. In line with this, EVs derived from cardiac stromal cells isolated from HF patients and introduced to a mouse model of MI resulted in impaired heart repair and deteriorated heart function [113]. On the contrary, EVs derived from healthy cardiac stromal cells enhanced heart repair and improved heart function [113]. More importantly, it seems feasible to transform the culprit EVs with therapeutic agents. In a diabetic cardiomyopathy animal model, overexpression of HSP20 (a downstream target of miR-320) in cardiomyocyte-derived EVs was able to enhance angiogenesis and improve heart function [129]. Further study is warranted to validate this EV “transformation” strategy in more preclinical animal models.

In contrast to cardiomyocyte-derived EVs, EVs released from cardiac progenitor cells (a group of cells capable of differentiating into cardiac cells upon stimulation with specific cues) are mostly beneficial, regardless of the microenvironment, they are generated in [105,130,131,132]. These EVs were reported to promote angiogenesis via their unique contents, mainly matrix metalloproteinases (MMPs) and extracellular matrix metalloproteinase inducer (EMPRINN) [130]. Several other studies showed that cardiac progenitor cells derived EVs contained a cluster of miRNAs that were pro-angiogenic and anti-fibrotic, and administration of these EVs in vivo was able to reduce infarct size and improved heart function in animals subjected to MI [131,132,133,134]. Interestingly, adult cardiac progenitor cells require a hostile environment, such as hypoxia, to produce efficacious EVs while the oxygen levels do not affect the efficacy of EVs derived from fetal cardiac progenitor cells [133]. The mechanism underlying this difference remains elusive.

### 4.3. Extracellular Vesicles Derived from Other Cell Types

EVs can be produced by various types of cells, with endothelial cells and platelets being the major sources in vivo. EVs may be released from cancer cells during certain medical treatment, such as microbubbles-assisted ultrasound, that assists in drug delivery [135]. In the context of CVD, endothelial EVs have been shown to promote angiogenesis and alleviate atherosclerosis, whereas platelet-derived EVs may either facilitate revascularization in the ischemic heart or deteriorate existing diseases (e.g., atherosclerosis) likely depending on the microenvironment [136,137,138,139]. EVs derived from cardiac fibroblasts that were subjected to angiotensin II could induce cardiomyocyte hypertrophy both in vitro and in vivo [140]. EVs derived from vascular smooth muscle cells are less explored, with one study showing their involvement in atherosclerosis-related vascular calcification [141]. EVs derived from immune cells have attracted increasing attention in recent years as it may modulate immune response and heart wound healing as summarized in recent reviews [142,143]. Intriguingly, a recent study showed that even plasma EVs from healthy adult humans or animals were able to protect the heart from injuries [144]. Despite the fact that the cellular origin of plasma EVs is not clear and plasma EVs are likely from multiple sources, this study raises an important question: Are there certain universal cardioprotective “factors” carried by EVs of different cellular origins?

## 5. Extracellular Vesicles as a Potential Drug Delivery System in CVD

The intrinsic therapeutic activity and the cell-to-cell communication roles of EVs have led to the concept of exploiting these vesicles as a drug delivery system (DDS) for targeted release of bioactive molecules. An ideal DDS aims to improve the pharmacological effect of a drug at the diseased site with minimum side effects [145,146]. Towards that purpose, several materials have been engineered into different sizes and shapes and exploited as potential DDS. Among them, vesicular carriers consisting of phospholipid bilayers (the so-called liposomes) were conceived as “synthetic mini-cells”, enabling several therapeutics to be incorporated, protected from metabolic inactivation and able to exert their effect(s) only upon release from their carrier [147,148]. Indeed, these membrane vesicles with nanoscale dimensions (which evade their premature recognition and removal from the bloodstream by the mononuclear phagocyte system) have the ability to encapsulate a wide variety of drug molecules (hydrophilic, hydrophobic, or amphipathic) either in their aqueous core or within their lipid bilayer. Being synthetic in nature, the properties of liposomes are highly tunable: Liposomes can be easily functionalized at their surface with different moieties to achieve different purposes (e.g., incorporation of ligands targeting receptors that are overexpressed in pathological conditions). Though to the best of our knowledge, there is no FDA-approved nano-formulation for the treatment of CVD [149,150], liposomes have been demonstrated to improve the therapeutic outcomes in several animal studies of CVD (Table 2).

Like other synthetic nano-particulate systems, liposomes take advantage of the enhanced permeability and retention (EPR) effect, which enables their passive accumulation at areas of highly vascular permeability, e.g., inflammatory sites in the ischemic heart [151,152]. However, liposomes have shown poor cellular uptake [153,154], as well as short blood circulation unless they are further modified through PEGylation. The main reason behind these phenomena is that liposomes are synthetic in nature. Hence, they lack any specificity towards target cells. Conversely, EVs, being the endogenous counterpart to liposomes, may offer an opportunity to overcome some of these limitations. Indeed, EVs have a natural phospholipid bilayer that enables them to retain loaded cargos in a similar fashion as liposomes [155]. More importantly, EVs contain surface proteins that can be recognized by target cells, enhancing their uptake and consequently increasing the efficiency of drug delivery into target cells. Furthermore, EVs have been shown to be more biocompatible, with a lower immunogenic potential and a higher stability in plasma than their synthetic counterparts [156].

Nonetheless, despite the vast potential of EVs to serve as a novel DDS, EVs also have limitations, such as rapid hepatic clearance [157]. Interestingly, the predominant accumulation and subsequent rapid clearance of EVs in the liver might be partly solved by modifying hepatic macrophages with the clinically approved antimalarial agent chloroquine [158]. Nonetheless, recent studies have tried to improve the targetability of EVs to the diseased sites in cardiovascular system through EV surface modification with specific peptides [159,160,161,162,163,164].

### 5.1. Atherosclerosis

Atherosclerosis is a common cause for a variety of CVD, including MI and ischemic stroke [165]. Due to the inflammatory nature and increased vascular permeability of atherosclerotic plaques, various nanoparticles have been designed to target atherosclerotic plaques for diagnosis or treatment purposes [166]. Despite obvious advantages (e.g., decreased dose with the same therapeutic effect) and substantial benefits (e.g., decreased number of unstable plaques) offered by the clinically used nanoparticles, their synthetic nature also creates certain limitations, such as potential toxicity in vivo [167]. In this regard, EVs may offer an attractive alternative.

Atherosclerosis is characterized by endothelial dysfunction at the predisposed sites along blood vessels. Therefore, the inflamed endothelial cells can become a target for drug delivery. An in vitro study demonstrated the potential of endothelial-derived EVs in delivering siRNA into primary endothelial cells isolated from mouse aorta, suggesting that EVs may be a natural DDS to deliver therapeutic nucleic acids to the inflamed endothelium in atherosclerosis [168]. A few atheroprotective miRNAs have been identified in EVs. miR-143/145, two miRNAs, known to prevent smooth muscle cell de-differentiation, were enriched in EVs derived from shear-stress stimulated endothelial cells [169]. Intravenous administration of those miR-143/145-rich EVs in ApoE^-/-^ mice fed with a high-fat diet reduced aortic fatty lesion by approximately two-folds and prevented atherosclerotic lesion formation [169]. Another example is miRNA-126 that was enriched in EVs derived from endothelial cells undergoing apoptosis [170]. In vivo studies demonstrated significant re-endothelization when the EVs were administered intravenously in a mouse model of carotid artery injury by electric endothelial denudation [170]. These studies suggest that enrichment of therapeutic miRNAs into EVs via genetic approaches can render EVs efficacious for atherosclerosis treatment. Interestingly, compared to atherosclerosis, the use of EVs as DDS is more extensively studied in MI and ischemic stroke.

### 5.2. Myocardial Infarction

MI is mainly caused by blockage of coronary arteries resulting in massive oxidative stress and inflammatory responses followed by cell death and irreversible heart tissue damage. Minimizing cardiac damage while repairing the injured cardiac tissue or regeneration of functional cardiomyocytes to replace the damaged tissue have always been the main challenges in the field. Apart from stem cell therapies and paracrine therapies using EVs of various cellular origins as discussed earlier, drug delivery using synthetic DDS, such as liposomes (Table 2) as well as EVs, have recently emerged as an attractive strategy to salvage the injured heart.

EVs, similar to synthetic DDS, can target the inflamed myocardium via the EPR effect due to their nanosize [134,177]. One approach of using EVs as a DDS is to load therapeutic compounds into EVs without surface modification. In a particular study, a cardioprotective miRNA, miR-93-5p, was loaded into EVs by genetic modification of EVs parental cells [178]. Intravenous administration of miR-93-5p-loaded EVs reduced myocardial damage and infarct size by inhibiting TLR4/NK-kB mediated inflammatory response and Atg7-mediated autophagy [178]. Two other similar studies using the same EVs but containing miR-126 or miR-146a showed similar treatment efficacy as of miR-93-5p-loaded EVs in a rat MI model [178,179,180]. However, in these studies, the targeting of EVs to the site of tissue inflammation mainly relied on the EPR effect. It is conceivable to improve organ-specificity of drug delivery by using a phage peptide display library and in vivo biopanning approach, an effective in vivo screening method to identify targeting peptides in specific diseases [181]. Through these methods, Zahid et al. identified a cardiac targeting peptide (CTP; APWHLSSQYSRT) that specifically targets the heart tissue with minimal uptake by other organs, including the liver [182]. By genetic fusion of this CTP with an EV surface protein Lamp2b, the targeting efficiency of EVs to the heart could be increased by 15% [160]. Although this peptide could direct the delivery of therapeutic protein or nucleic acids in EVs to the heart irrespective of the EPR effect, it could not differentiate between healthy or damaged cells, posing a potential risk for undesired side effects to the healthy heart cells. Instead, a peptide that specifically targets infarcted heart tissue would be more attractive. With the same approach as Zahid et al. [182], Kanki et al. identified in a rat IR injury model a cardiac homing peptide (CHP; CSTSMLKAC) that could specifically target ischemic myocardium [183]. To enhance targeting efficiency of EVs to ischemic myocardium, CHP was engrafted onto EV surface. Similarly, cardiosphere-derived stem cells generated EVs that were chemically conjugated with CHP could increase the accumulation of EVs at the injured site of the myocardium in a rat myocardial IR injury model [159]. Compared with non-conjugated EVs, CHP-conjugated EVs exerted better therapeutic efficacy against MI in terms of myocardial infarct size, fibrosis, angiogenesis, and cardiac function [159]. By genetic fusion with an EV surface protein Lamp2b, CHP was decorated on the surface of murine MSC-derived EVs [161]. Similar to the CHP-conjugated EVs, these genetically engineered EVs were able to target the ischemic heart more efficiently as compared to non-modified EVs, and therefore showed better therapeutic efficacy against MI-induced cardiac dysfunction [161].

### 5.3. Stroke

Stroke, in particular, ischemic stroke, is mainly caused by narrowing or blockage of cerebral vessels in the event of atherosclerotic plaque rupture. Therefore, stroke is classified as a type of CVD [2]. Different from MI, systemic drug delivery is often impeded by the blood–brain barrier (BBB) [184]. In contrast to other DDS, EVs, in particular, exosomes, seem to be able to pass this BBB. In a zebrafish brain cancer model, it was shown that exosomes derived from brain endothelial cells could pass through the BBB and deliver anti-cancer drugs to the brain [185]. In a rodent stroke model, intravenous administration of MSC-derived EVs containing miR-17-92 significantly reduced the neurological deficits compared to liposomal formulation [186]. These studies suggest that native EVs are able to cross the BBB and deliver neuroprotective drugs, such as miRNAs, to the ischemic brain. However, the targeting efficiency is low as EVs are also being taken up in other organs, such as the liver. To increase the delivery specificity to the brain, Alvarez-Erviti et al. designed an elegant genetic engineered EV system by fusion of a neuron-specific RVG peptide (YTIWMPENPRPGTPCDIFTNSRGKRASNG) with an EV surface membrane protein, Lamp2b [187]. Using these engineered EVs, the authors delivered a siRNA of a therapeutic target in Alzheimer’s disease and successfully knocked down this gene in a variety of brain cells, while no significant uptake of these EVs was observed in other organs [187]. More attractively, Tian et al. functionalized MSC-derived exosomes by click chemistry with a peptide (RGDyK) that exhibits high affinity to integrin αvβ3 in reactive cerebral vascular endothelial cells after brain ischemia [163]. The functionalized exosomes could efficiently cross the BBB and target the ischemic brain tissue [163]. Using these surface functionalized exosomes, the same research group has delivered EV-loaded curcumin (an anti-inflammatory compound) [163] and miR-210 [164] to the ischemic brain and achieved significant treatment efficacy. Further validation of these findings in large animal models or first-in-man clinical trial is warranted.

## 6. Extracellular Vesicles as a Theranostic Platform

Continuing advances in EV technology, as described in previous sections, make EVs a novel and attractive DDS. In addition to therapeutics, diagnostic agents can also be loaded in EVs to track the injected EVs. The combination of therapeutic and imaging broadens the application of EVs as a theranostic platform [188,189,190]. Among such diagnostic agents reported to date, magnetic nanoparticles are commonly used as an imaging agent. Magnetic nanoparticles have several advantages over other modalities, and they can be used as magnetic resonance imaging contrast agents [191,192]. The most widely used magnetic nanoparticles are iron-oxide nanoparticles (IONPs) and superparamagnetic iron-oxide nanoparticles (SPIOs) [193]. To generate theranostic EVs, diagnostic agents and therapeutic molecules can be incorporated into EVs by loading the two into EV parent cells or directly loading them into EVs by electroporation [194,195,196]. Although the theranostic EVs were initially designed for advanced imaging of anti-cancer treatment, this technology platform, in principle, can be used for CVD. In fact, the feasibility of using SPIOs to enhance the magnetic resonance imaging of atherosclerotic plaques and the macrophages surrounding these plaques has been demonstrated in human subjects [197]. The enhanced imaging allows clinicians to evaluate the efficacy of the treatment and to identify vulnerable plaques in the arteries [197]. Given the various advantages of EVs over synthetic nanoparticles as a DDS and the potential biomarkers identified in EVs as well as the intrinsic therapeutic activity of EVs discussed earlier, EVs seem to offer a unique theranostic platform with great potential. With the development of the large-scale production of EVs or EV mimetics from autologous cells, personalized theranostic EVs may be considered for precision medicine.

## 7. Limitations and Future Perspective

**Figure 2:**
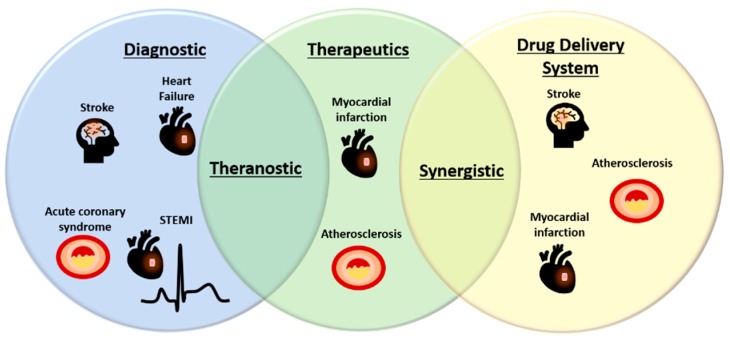
Potential applications of EVs in diagnostic, therapeutic, and drug delivery in CVD. Increasing novel biomarkers identified in EVs have made them as attractive alternative sources of biomarkers for a variety of CVD. On the other hand, EVs also bear intrinsic therapeutic activities and great potential to deliver drugs or therapeutic compounds and therefore, may offer synergistic treatment effects. Endogenous EV biomarkers and/or loaded diagnostic agents may be housed together with therapeutic compounds to create theranostic EVs for CVD treatment. STEMI, ST-elevation myocardial infarction.

EVs offer exciting promise for biomarker discovery, therapeutic and drug delivery in CVD (Figure 1 and Figure 2). However, several limitations of EVs should be taken into consideration and need to be overcome to enable their broad clinical applications. In biomarker studies, despite a variety of EV miRNAs and proteins being identified for CVD diagnosis or prognosis, the sample size is often small and mostly in single cohorts. As such, validation of potential EV biomarkers in multi-center clinical cohorts is required. Setup of parallel bio-banks with standardized protocols could facilitate the validation studies [198]. To develop clinically useful EV-based therapeutics, drug delivery systems or theranostics, the pharmacokinetics of EVs in vivo is critical but largely unknown. The non-specific delivery of EVs to undesired tissues also hinders the potential application of EVs as therapeutics or drug delivery systems. To achieve the effective dose for a desired therapeutic effect, either from EV intrinsic activity or from EV-loaded compounds, sufficient doses of EVs reaching the disease sites are necessitated [159,161]. However, there is a lack of studies in evaluating the toxicity of EVs at higher doses. In addition, the variability of methods for isolating EVs makes it difficult to validate reported findings for further development. To date, there is no universally accepted and validated protocol for isolating the subpopulations of EVs [6]. The various sub-populations of EVs have different protein and lipid contents, and this may affect the therapeutic function of these vesicles [199]. Furthermore, the cell preparation techniques for producing EVs may affect the EV contents, thereby compromising the reproducibility of results observed in preclinical settings [199].

Another general limitation for the application of EVs is the relatively cumbersome production process. The yield of EVs from cell culture or biofluids is typically very low; hence, bulk production of EVs is needed. Recently, researchers have been trying to produce “EVs” in bulk by engineering approaches (referred to as EV mimetics in this review). These EV mimetics are at the nanoscale, commonly referred to as exosome mimetic nanovesicles [200], biomimetic nanovesicles [201], or cell-derived nanovesicles [202]. These EV mimetics are usually produced by subjecting the parent cells through serial extrusion with filters of progressively smaller sizes (100–400nm). These approaches could produce much higher yields of EV mimetics in a shorter time; and importantly, the resultant EV mimetics retain comparable therapeutic activity to native EVs [203]. While engineering approaches may streamline the production process and improve yield, these EV mimetics generally have lower drug loading efficiency [204]. Further modification of the EV mimetics has been carried out by fusing these mimetic vesicles with synthetic components to create hybrid EVs, which are armed with both intrinsic EV properties and high loading efficiency close to liposomal formulations [154]. Hybridization of EV mimetics with synthetic nanomaterials may offer a novel opportunity to develop EV-based drug delivery systems and theranostics, and therefore, may promote their translation into clinical practice for CVD management.

## Figures and Tables

**Table 1:** Summary of extracellular vesicle contents as diagnostic or prognostic biomarkers in CVD.

Disease	Sample Size	Source	Methods of Isolation	EVs Characterized	Screening Method	Quantification Method	Analytes Measured	Significant Candidates	Potential Application	Ref.
Acute Ischemic Stroke	50	Serum	Precipitation	TEM; Western blot	Based on previous reports	RT-qPCR	miR-134	miR-134	Diagnosis and prognosis	[59]
Acute Ischemic Stroke	50	Serum	Precipitation	TEM; Western blot	Based on previous reports	RT-qPCR	miR-223	miR-223	Diagnosis and prognosis	[60]
Heart Failure	31	Plasma	Precipitation	Not available	Based on previous reports	RT-qPCR	miR-221, miR-15a, miR-122, miR-21, miR-29, miR-30d, miR-133a, miR-425, miR-744	miR-21, miR-425, miR-744	Diagnosis, prognosis and therapeutic candidates	[62]
Acute Heart Failure	28	Serum	Precipitation	TEM; Western blot; DLS	Based on previous reports	RT-qPCR	miR-92b-5p, miR-192-5p, miR-320a	miR-92b-5p	Diagnosis	[63]
Acute Myocardial Infarction	21	Serum	Precipitation	Western blot	miRNA profiling through TaqMan Human MicroRNA Array	RT-qPCR;	377 miRNAs	miR-192, miR-194, miR-34a	Prognosis	[64]
Atrial fibrillation	40	Serum	Precipitation	TEM; NTA; Western blot	miRNA microarray	RT-qPCR	miR-107, miR-320d, miR-103a, miR-486, miR-let-7b	miR-107, miR-320d, miR-103a, miR-486, miR-let-7b	Diagnosis, prognosis	[57]
Hyper-tension	22	Serum	Ultra-centrifugation	TEM; Immune-gold labelling	Nanostring nCounter^®^ platform	Nanostring nCounter^®^ platform	800 miRNAs	miR-199a/b, miR-223–3p	Early diagnosis	[67]
Obesity	883	Plasma	Ultra-centrifugation	TEM; FACS; NTA	miRNA screening through QuantStudio™12 K FlexOpenArray^®^ Platform	miRNA screening through QuantStudio™12 K FlexOpenArray^®^ Platform	754 miRNAs	miR-let-7c-5p, miR-106a-5p, miR-143-3p; miR-185-5p, miR-218-5p; miR-331-3p, miR-642-5p, miR-652-3p, miR-99b-5p	Early diagnosis	[68]
Coronary Artery Disease	21	Plasma	Column-based system (Exo-spin Mini Columns)	NTA; TEM; Western blot	Based on previous reports	RT-qPCR	14 miRNAs	miR-1, miR-24, miR-133a, miR-133b, miR-210	Diagnosis	[73]
Acute Coronary Syndrome	475	Serum	Precipitation	TEM; FACS; NTA; Western blot	Differential quantitative proteomics and ingenuity pathway analysis	Multiplex immunoassay	polygenic immunoglobulin receptor (pIgR), cystatin C, and complement factor C5a	pIgR, cystatin C	Diagnosis	[40]
Acute Myocardial Infarction	25	Plasma	Ultra-centrifugation	FACS; TEM; DLS	2D-DIGE; LC-MS/MS; MALDI-TOF coupled with ingenuity pathway analysis	2D-DIGE; Western blot	25 ORFs derived from 102 differentially regulated proteins spot identified through 2D-DIGE	A2-macroglobulin isoforms, Fibrinogen, Viperin	Diagnosis and therapeutic targets	[80]
Vascular Disease	1060	Plasma	Precipitation	TEM; FACS; NTA; Western blot	Differential quantitative proteomics and ingenuity pathway analysis	Multiplex immunoassay	Cystatin C, Serpin G1, Serpin F2, and CD14	Cystatin C, Serpin F2, and CD14	Prognosis	[41]
Dyspnea and Heart Failure	404	Plasma	Sequential density precipitation	TEM; Western blot	Based on previous study	Multiplex immunoassay	Cystatin C, Serpin G1, Serpin F2, and CD14	CD14, SerpinG1, and SerpinF2	Prognosis	[42]
Vascular Disease and Obesity	1060	Plasma	Precipitation	TEM; FACS; NTA; Western blot	Differential quantitative proteomics and ingenuity pathway analysis	Multiplex immunoassay	Cystatin C, Serpin G1, Serpin F2, and CD14	Cystatin C, CD14	Prognosis and therapeutic target	[81]
Obesity	22	Plasma	Ultra-centrifugation	TEM; FACS; NTA; Western blot	2D-DIGE-based proteomic approach	2D-Western blot	Entire Proteome	C3, C4, Fibrinogen, Adiponectin	Early diagnosis	[82]

**Table 2:** Selected examples of liposomal formulation used in the in vivo model for treatment of CVD.

Therapeutics Encapsulated	In vivo Model	Outcome	Ref.
Simvastatin/Alendronate	Carotid-injured rat model	Suppressed neointimal formation	[171,172]
Nitric oxide	Carotid-injured rabbit model	Suppressed neointimal formation	[173]
Berberine	Myocardial infarction murine model	Improvement of cardiac function	[152]
VEGF	Myocardial infarction rat model	Improved cardiac functions	[174]
Cyclopentenone prostaglandin	Hyperlipemic diet atherosclerotic murine model	Vascular injuries recovery	[175]
Fumagillin	Hyperlipemic diet atherosclerotic murine model	Reduced atherosclerotic lesions	[176]

## Abbreviations

2D-DIGE	two-dimensional difference gel electrophoresis
ACS	acute coronary syndrome
ATG7	autophagy related 7
BBB	blood–brain barrier
CABG	coronary artery bypass graft
CHP	cardiac homing peptide
CTP	cardiac targeting peptide
CVD	cardiovascular disease
DDS	drug delivery system
DLS	dynamic light scattering
DNA	deoxyribonucleic acid
EMPRINN	extracellular matrix metalloproteinase inducer
EPR	enhanced permeability and retention
ESCRT	endosomal sorting complex required for transport
EVs	extracellular vesicles
FACS	fluorescence-activated cell sorting
FDA	food drug administration
GC	gas chromatography
HF	heart failure
HSP20	heat shock protein 20
HSP60	heat shock protein 60
IONPs	iron-oxide nanoparticles
iPS	induced pluripotent stem cells
IR	ischemic reperfusion
ISEV	international society of extracellular vesicles
KO	knock-out
LC	liquid chromatography
lncRNA	long-non-coding RNA
MALDI-TOF	matrix-assisted laser desorption/ionization-time of flight
MI	myocardial infarction
miRNA	microRNA
MMPs	matrix metalloproteinases
mRNA	messenger RNA
MS	mass spectrometry
MSC	mesenchymal stem cells
NF-κB	nuclear factor kappa-light-chain-enhancer of activated B cells
NIHSS	national institutes of health stroke scale
NTA	nanoparticle tracking analysis
ORF	open reading frame
PE	phosphatidylethanolamine
pIgR	polygenic immunoglobulin receptor
PS	phosphatidylserine
RISC	RNA-induced silencing complex
RNA	ribonucleic acid
RT-qPCR	reverse transcription quantitative polymerase chain reaction
siRNA	small interfering RNA
SPIOs	superparamagnetic iron-oxide nanoparticles
STEMI	st-elevation myocardial infarction
TEM	transmission electron microscopy
TLR4	toll-like receptor 4
TNF-α	tumor necrosis factor-alpha
tRNA	transfer RNA
TSG101	tumor susceptibility gene 101
VEGF	vascular endothelial growth factor
